# The Future of Breast Cancer Treatment: From Conventional Wisdom to Nanotechnology-Enabled Precision

**DOI:** 10.3390/ijms27052109

**Published:** 2026-02-24

**Authors:** Neetika Kimta, Malak A. Majdalawieh, Amin F. Majdalawieh

**Affiliations:** 1 School of Biological and Environmental Sciences, Shoolini University of Biotechnology and Management Sciences, Solan 173229, India; neetikimtam5@gmail.com; 2 Department of Biology, Chemistry and Environmental Sciences, College of Arts and Sciences, American University of Sharjah, Sharjah P.O. Box 26666, United Arab Emirates; g00104441@aus.edu; 3 Advanced Biosciences and Bioengineering Research Center, American University of Sharjah, Sharjah P.O. Box 26666, United Arab Emirates; 4 Bioinformatics and Computational Biology Research Group, American University of Sharjah, Sharjah P.O. Box 26666, United Arab Emirates

**Keywords:** breast cancer, conventional therapy, nanotechnology, nanorobotics, targeted drug delivery

## Abstract

Breast cancer remains a leading cause of mortality among women worldwide, presenting significant treatment challenges due to its aggressive nature and lack of targeted therapies. Traditional treatments, including surgery, radiation, and chemotherapy, have improved survival rates. However, limitations such as drug resistance and adverse side effects persist. Recent advancements in nanotechnology offer promising avenues for enhancing breast cancer treatment by improving drug delivery, increasing therapeutic efficacy, and minimizing systemic toxicity. This review explores breast cancer’s pathophysiology and molecular mechanisms, evaluates current diagnostic and therapeutic strategies, and discusses emerging innovations in nanotechnology and integrative medicine. Recent studies have demonstrated the efficacy of green nanotechnology in transforming Ayurvedic medicine into scientifically credible treatments. These integrative approaches not only enhance the therapeutic potential of traditional medicine but also offer a pathway to overcome challenges associated with conventional cancer treatments. The convergence of nanotechnology and traditional medical systems holds transformative potential in breast cancer therapy. By leveraging the unique properties of nanoparticles and nanorobotics, it is possible to develop more effective, personalized, and less toxic treatment modalities. Future research should optimize these integrative approaches, conduct rigorous clinical trials, and elucidate the underlying mechanisms to fully realize their potential in combating breast cancer.

## 1. Introduction

Cancer is a disease where cells in the body multiply uncontrollably. Malignant tumors (tri-dosage) are exceedingly detrimental since these bodily control systems lose their mutual coordination, hence failing to avert tissue destruction, which culminates in a fatal sick state [1]. Molecular heterogeneity is a hallmark of breast cancer, the most common disease in women. Approximately 2 to 2.5 million people globally are affected by breast cancer (BC) every year, making it a prevalent type of disease. When it comes to female cancers, breast cancer is well out in front in India.

According to the World Health Organization, approximately 170,000 women are impacted by this disease, representing an increase of around 14% in the total cancer cases within the country. Luminal A, luminal B, HER2 type, and estrogen (ER) and progesterone (PR) positive breast cancers are among the many molecular subtypes that may be identified [2]. Out of all the subtypes described, the majority, over 70%, are ER/PR positive [3]. Twenty percent of breast cancers are triple-negative breast cancer (TNBC), meaning they do not express HER2, ER, or PR [4]. Gene mutations and genomic instability in several tumor suppressor genes may contribute to the increased metastatic rate [5]. The percentage of cases with TNBC ranged from around 7% to 28% in different nations throughout the world, with the greatest prevalence reported in India [6].

Developments in multimodal treatment have brought about improved prognoses for cure in around 70–80% of patients. However, advanced sickness is considered incurable given the present state of medicine [7]. The chance of developing breast cancer may be increased by several things, including one’s age, sex, estrogen levels, family history, genetic mutations, and lifestyle choices [5]. The incidence of breast cancer is one hundred times higher in females than in males, and the vast majority of breast cancer cases are diagnosed in women [8].

The advancement of mammography has significantly enhanced the chances of early breast cancer detection. Breast cancer death rates dropped by 30% in women aged 50–69 who were screened with a mammography machine once a year, according to randomized clinical studies [9]. Mutations in cancer cells’ biochemical traits, such as changes to the apoptosis regulatory process, multidrug resistance-associated protein (MRP), enzyme activity (e.g., topoisomerase), or transport mechanisms (e.g., the P-glycoprotein efflux system), are the root causes of resistance [10]. There is a wide variety of materials that may be created using nanoparticles.

Consequently, the advancement of successful treatments for invasive breast cancer, especially in cases of highly metastatic disease, continues to be a crucial focus. The transformation of traditional therapies into innovative drug delivery systems, such as nanoparticles, has the potential to enhance the pharmacokinetic and pharmacodynamic characteristics of anticancer medications [11]. Additionally, specific nanoparticles have demonstrated the ability to address multidrug resistance (MDR), a significant challenge in chemotherapy. Nanotechnology offers many advantages over traditional medicine, such as the ability to modify surfaces, increase drug entrapment efficiency, lengthen circulation half-lives, and engage in active or passive targeting. Chemotherapy frequently fails to deliver a targeted and comprehensive response in patients with terminal cancer (metastatic stage), potentially resulting in the patient’s demise [12].

The efficacy of nanoparticles is contingent upon their capacity to target and diminish or eradicate tumors while preserving the integrity of normal cells. During systemic distribution throughout the body, drug-containing nanoparticles have longer retention periods inside tumor masses compared to normal tissues. Tumor cells retain nanoparticles to a greater extent due to impaired lymphatic drainage and damaged blood vessel integrity. The phenomenon in question is known as the enhanced permeability and retention (EPR) effect [13].

Breast cancer remains one of the most prevalent and complex diseases worldwide, necessitating continued research and innovative approaches for its diagnosis, treatment, and understanding. This review article attempts to provide a complete and updated overview of breast cancer, incorporating the newest results across many fields. This review seeks to encapsulate the most comprehensive information available on a multidisciplinary approach, exploring pathophysiology, molecular mechanisms, genomic insights, tumor biology, and current diagnostic strategies in tandem with evolving therapeutic options, such as conventional therapies, nanotechnology, and nanorobotics. However, discoveries about its pathophysiology, molecular mechanisms, and genomic landscape are opening the door to more individualized and accurate treatments very quickly. Conventional therapies remain integral to breast cancer management, yet the integration of nanotechnology and nanorobotics holds transformative potential, offering new avenues for early detection, targeted drug delivery, and minimized side effects.

## 2. Pathophysiology

**Figure 1:**
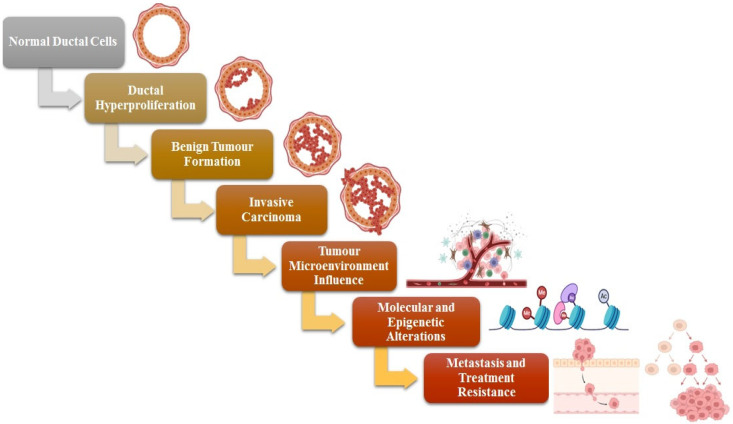
Mechanism of initiation and development of breast cancer, illustrating the transition from normal ductal epithelial cells to hyper-proliferation, tumor formation, and carcinoma development. The figure also highlights the influence of the tumor microenvironment, the accumulation of genetic and epigenetic alterations, and the emergence of treatment resistance during disease progression.

Breast tumors originate from ductal hyperproliferation and can advance to benign tumors or metastatic carcinomas when persistently influenced by various carcinogenic factors. All types of breast cancer originate from the terminal ductal lobular unit. Invasive breast cancer encompasses a varied array of lesions that can be classified according to their histological features, as illustrated in Figure 1. Approximately 80% of breast cancer cases are classified as ductal carcinomas, representing the most common type of lesion and exhibiting significant diversity within this category. Lesions that are negative for E-cadherin, including lobular carcinoma, represent the second most prevalent type, accounting for 10–20% of cases. Macrophages and stromal influences represent two significant components of the tumor microenvironment, which play essential roles in the progression and dissemination of BC [14,15]. Cancer stem cells, commonly referred to as CSCs, represent a distinct category of malignant cells identified within tumors, associated with processes such as tumor initiation, evasion, and recurrence. This small group of cells possesses the ability to self-renew and demonstrates resistance to standard therapies such as chemotherapy and radiation [16]. Stem cells and progenitor cells, which are present in normal tissues, might be their sources. Contrary to popular belief, breast cancer stem cells (BCSCs) are not likely generated from basal stem cells but rather from luminal epithelial progenitors [17]. Wnt, Notch, Hedgehog, p53, PI3K, and HIF are among the signaling pathways that control the invasion, proliferation, and self-renewal processes in BCSCs [18].

### 2.1. Mechanism

While the exact mechanism behind the initiation of breast cancer remains unclear, significant research has been conducted to elucidate the origins and progression of breast cancer, as well as to characterize its molecular features. A spectrum of morphological abnormalities and genetic changes exists, spanning from healthy glands to cancer. Evidence suggests that breast cancer advances through two separate molecular pathways, primarily linked to estrogen receptor expression as well as tumor grade and proliferation. Furthermore, certain elements of the pathophysiology associated with both inherited and sporadic breast cancer have been elucidated through the identification of genes that increase susceptibility to the disease [19]. The first route, called the low-grade-like pathway, has a gene expression signature (GES) that is mostly linked to the ER phenotype, diploid or near-diploid karyotypes, low tumor grade, and a gain of 1q, a loss of 16q, and an infrequent amplification of 17q12. Here you may find the luminal A group and, to a lesser extent, the luminal B group. Loss of 13q, increase in chromosomal area 11q13, and amplification of 17q12 (including *ERBB2*, the gene encoding *HER2*) characterize the second route, which is called the high-grade-like pathway. On top of that, genes related to the cell cycle and cell proliferation have an expression signature [20]. Breast tumors that are *HER2*-positive and triple-negative fall into the category of high-grade tumors, which are covered by this route [21].

#### 2.1.1. Molecular Alterations

In a study of early breast cancers [22], the tumor cells exhibited the most frequent mutations and/or amplifications in the following genes: *TP53* (41% of tumors), *PIK3CA* (30%), *MYC* (20%), *PTEN* (16%), *CCND1* (16%), *ERBB2* (13%), *FGFR1* (11%), and *GATA3* (10%). Encoding cell-cycle modulators that may activate or repress processes is the responsibility of these genes, thereby sustaining cell proliferation and/or averting apoptosis. They also serve to inhibit activated oncogenic pathways such as *MYC*, *HER2*, and FGFR1, as well as elements that have lost their regulatory control, like *PTEN*. Most breast cancers arise from multiple low-penetrant mutations that accumulate over time, as the majority of mutations affecting the 100 suspected breast cancer drivers are quite rare [23].

#### 2.1.2. Epigenetic Alterations

The development and metastasis of breast cancer are influenced by epigenetic changes. Focal hypermethylation at some loci may be seen in breast cancer genes, which can suppress genes and cause genetic instability by blocking genes that normally repair DNA. Conversely, chromosomal instability, gene activation, and oncogene overexpression might occur as a consequence of global hypomethylation. Further epigenetic mechanisms encompass nucleosomal remodeling, alterations in chromatin structure that inhibit gene expression, and modifications to histone tails resulting from DNA methylation. These modifications are possibly targetable, reversible, and facilitated by enzymes [24].

#### 2.1.3. Hormone Receptors

An important determinant of sporadic breast cancer is hormone exposure. Evidence for estrogen’s function in breast cancer promotion comes from its binding to the nucleus-based estrogen receptor, which is encoded by the ligand-activated transcription factor ESR1. Throughout the stages of adolescence, menstruation, and pregnancy, when the organ is active, hormones facilitate the growth of the breasts. An uneven ratio of estrogen to progesterone during the menstrual cycle encourages cellular proliferation and could result in the buildup of DNA damage. The replication process that occurs in each cycle might cause the repair mechanism to become faulty, resulting in mutations in pre-malignant cells and, ultimately, in malignant cells. At this point in the cancer development process, estrogen promotes the proliferation of these cells and the establishment of stromal cells. By attaching to estrogen response elements in the promoter regions of certain genes, the estrogen receptor may activate and change gene expression. Furthermore, in the absence of estrogen [25], extracellular cues may promote ER production and activation. In addition, proteins like growth factor receptors may be directly engaged with by the endoplasmic reticulum (ER), which in turn enhances the production of genes linked to cell survival and proliferation [26]. Medications that either reduce estrogen’s effects on breast tissue (like Tamoxifen) or block estrogen synthesis (like aromatase inhibitors) are the mainstays of hormone-sensitive breast cancer therapy. Aromatase inhibitors may lead to osteoporosis due to the interaction of estrogen with bone, similar to the effects observed during menopause. Tamoxifen, conversely, inhibits osteoporosis by functioning on the bone similarly to estrogen.

#### 2.1.4. Immune Involvement

The complex setting in which breast cancer develops comprises many kinds of benign cells and the extracellular matrix, which both mechanically supports and allows paracrine cellular interactions. There are a variety of cell types seen in the breast cancer microenvironment, the most common of which are cancer-associated fibroblasts. Other cells of the leukocyte lineage include macrophages, lymphocytes, and myeloid-derived stromal cells. The majority of these cells are involved in the immunological response [27,28]. Breast cancer prognosis and neoadjuvant treatment efficacy are both enhanced by the presence of tumor-infiltrating lymphocytes, which indicate the strength of the immune response inside the tumor microenvironment [29].

## 3. Genomics

### 3.1. Genes Related to Breast Cancer

Breast cancer has been linked to a wide variety of genes. Tumors develop and spread due in large part to mutations in oncogenes and anti-oncogenes, as well as aberrant amplification. The breast cancer risk is linked to *BRCA1* and *BRCA2*, which are important anti-oncogenes. Some chromosomes have the *BRCA1* gene, whereas others carry the *BRCA2* gene on chromosome 13q12. They both code for proteins that inhibit tumor growth. Death is the end outcome of *BRCA1* loss-related dysregulation of cell cycle checkpoints, aberrant centrosome duplication, genomic instability, and so on [30,31]. In a mechanism that is reliant on E2F, the “pocket proteins,” which include the retinoblastoma protein, p130, and p107, suppress the production of *BRCA1*. Based on what we know so far, the *BRCA1* gene can control its expression via interactions between its promoter and a loop that includes its introns and terminator regions [32]. Breast cancer risk is substantially elevated in those who inherit harmful mutations in the *BRCA1* or *BRCA2* genes. Mutations in *BRCA1/2* are inherited via an autosomal dominant pattern, even while the second allele is normally functioning.

*HER2*, or *c-erbB-2*, is a significant oncogene linked to breast cancer. In humans, it may be found at 17q12 on the long arm of chromosome 17 [33]. The *HER2* gene is primarily activated by two processes: gene amplification and rearrangement. Initiating downstream signaling pathways, the HER2 protein joins forces with other ligand-bound Epidermal Growth Factor Receptor (EGFR) family members, namely Her3 and Her4, to create heterodimers [34]. The HER family includes the tyrosine kinase class of proteins.

In humans, the *EGFR* gene is located on the short arm of chromosome 7 (7p12). It is also known as c-erbB-1 or Her1. The cell surface glycoprotein EGFR, belonging to the tyrosine kinase family, is activated through its interaction with various proteins such as betacellulin, amphiregulin, TGF-α, and EGF. To encourage cell invasion, proliferation, angiogenesis, and protection against apoptosis, EGFR triggers downstream signaling pathways such as PI3K, Ras-Raf-MAPK, and JNK [35]. Overexpression of EGFR was seen in more than 30% of instances of inflammatory breast cancer (IBC), a particularly aggressive subtype of breast cancer. The prognosis was worse for patients with EGFR-positive IBC compared to those with EGFR-negative tumors [36]. Breast cancer, whether it is inherited or develops on its own, is influenced by genetic mutations [37]. Acquired mutations in the *PTEN* gene account for about 10% of breast cancer cases in humans, whereas p53 mutations account for around 40% of instances. Between 5 and 10% of breast cancer cases are attributed to high-penetrance cancer genes.

### 3.2. Tumor Biology

The characteristics of metastatic disease, including its timing and locations, are shaped by the intrinsic classification. Luminal A tumors, similar to luminal B and HER2-negative tumors, exhibit a propensity for late recurrence, often occurring five years post-initial diagnosis, and are particularly associated with bone and lymph node involvement. Triple-negative breast cancers frequently give rise to brain and visceral (lung) metastases and are susceptible to early recurrences, typically occurring within 2 to 3 years following the initial diagnosis. The outlook for HER2-positive breast tumors has improved with the introduction of anti-HER2 targeted therapies; however, numerous cases manage to bypass treatment by metastasizing to the brain. In Western countries, it is estimated that 20–30% of patients may experience a metastatic recurrence [38,39]. The specific traits of breast cancer tumors that lead to metastasis remain uncertain. Additionally, although certain research efforts are investigating treatments such as aspirin and metformin to prevent metastatic recurrence, the results remain largely inconclusive.

## 4. Diagnosis

The goal of population screening is to identify treatable illnesses in their early stages, utilizing a non-invasive, accurate, and user-friendly test. Population screening for breast cancer with mammography is an additional preventative measure that aims to find the illness early so that appropriate treatment options may be considered. An anticipated reduction in more intensive treatment is expected due to the enhanced early diagnosis of breast cancer through screening [40,41]. The recommended approach for screening individuals with *BRCA* mutations and women at a notably high lifetime risk of breast cancer involves the integration of MRI and mammography, enhancing the sensitivity of screening for those with *BRCA1* and *BRCA2* mutations [42].

**Figure 2:**
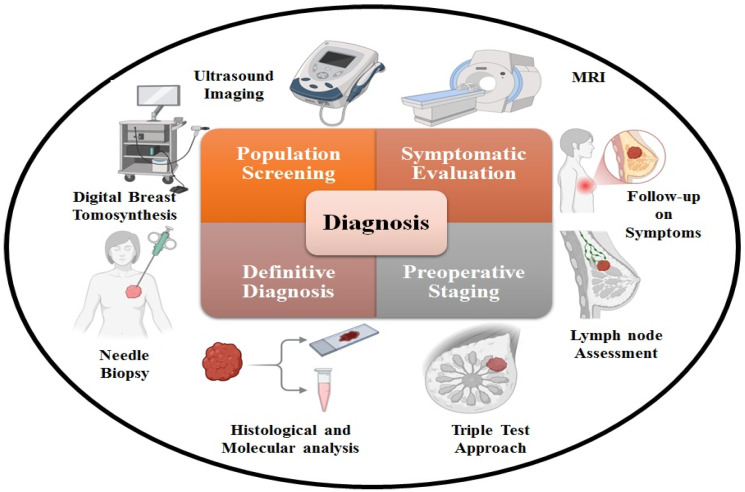
Breast cancer diagnosis process.

A diagnostic approach known as the triple test encompasses a clinical assessment, imaging techniques (commonly mammography and ultrasonography), and a needle biopsy to identify breast cancer [43]. To determine if a patient has breast cancer or a benign disease like fibroadenoma, or even typical breast alterations that may be safely treated with follow-up and perhaps avoid surgery, a precise evaluation is crucial **(**Figure 2**)**. When it comes to young women, ultrasonography is the go-to imaging tool for evaluating localized symptoms, identifying and characterizing abnormalities found during screenings, and, more and more, for image-guided percutaneous biopsies. Women who are suspected of having breast cancer may undergo characterization and biopsy of their axillary lymph nodes through breast ultrasonography [44]. Moreover, preoperative MRI is employed selectively to stage newly identified diseases; however, this method is subject to debate due to the limited evidence supporting its effectiveness in enhancing patient clinical outcomes [45]. Nonetheless, for the preoperative assessment of newly identified invasive lobular tumors, MRI is advised [46].

## 5. Risk Factors

### 5.1. Aging

**Figure 3:**
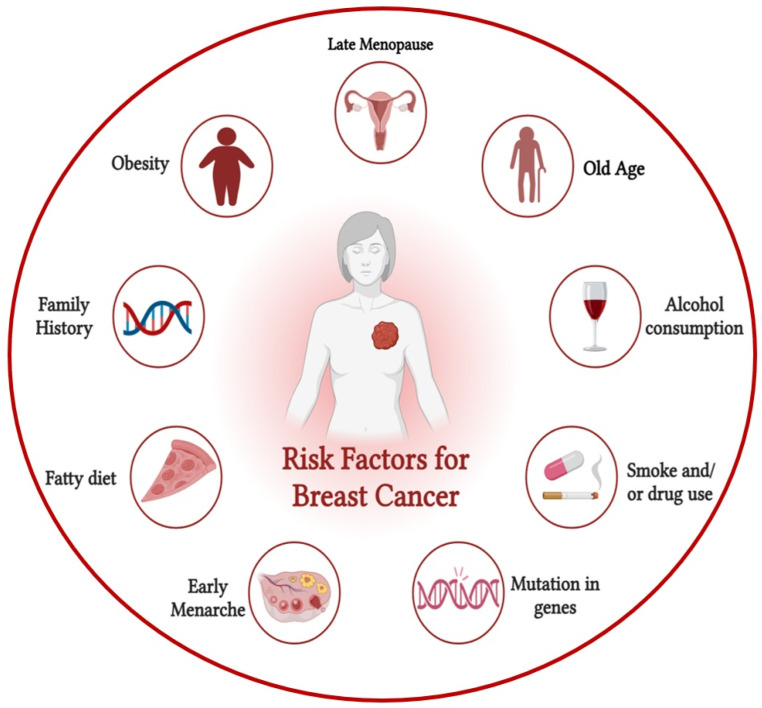
Various risk factors for breast cancer.

There is a substantial association between the incidence of the illness and rising age, making age a key risk factor for breast cancer, alongside sex, as demonstrated in Figure 3. 193% and 72% of the breast cancer fatalities in the US in 2016 were in women aged forty to sixty, respectively [8]. As a result, women aged 40 and above are required to secure a mammography screening beforehand. The familial context contributes to approximately 25% of all instances of breast cancer [47]. Hereditary breast cancer risk factors include abnormalities in genes linked to the illness, such as *BRCA1* and *BRCA2*.

### 5.2. Reproductive Factors

Factors related to reproduction, such as poor parity, delayed menopause, early menstrual start, and advanced maternal age, may impact the likelihood of breast cancer. Delaying menopause for even one more year raises the risk of breast cancer by 3%. There is a 5–10% decrease in the incidence of breast cancer for every extra birth or every year that menarche is delayed [48,49]. There has been a recent cohort study in Norway that shows a hazard ratio (HR) of 1.54 for first births at an advanced maternal age (≥35 years) compared to an early maternal age (<20 years) [50].

### 5.3. Estrogen

The use of either endogenous or exogenous estrogens increases the likelihood of breast cancer. The ovaries produce natural estrogen in women who have not yet gone through menopause, and removing them may lower the risk of breast cancer [51]. The two most common ways to obtain estrogen from outside sources are oral contraceptives and hormone replacement treatment (HRT). Hormones such as exogenous estrogen are given to women who are menopausal or postmenopausal as a component of hormone replacement therapy (HRT). Numerous studies have established a connection between the use of HRT and a heightened risk of breast cancer [52]. Research indicates that discontinuing hormone replacement therapy for two years significantly lowers the likelihood of developing breast cancer [53]. Among breast cancer survivors who take hormone replacement treatment, the recurrence rate is equally raised, and the hazard ratio for a new breast tumor is 3.6 [54].

### 5.4. Lifestyle

Contemporary lifestyle choices, such as binge drinking and excessive fat consumption, can elevate the risk of breast cancer. There is some evidence that alcohol consumption may increase blood levels of estrogen-related hormones and activate the estrogen receptor pathways. The contemporary Western diet contains excessive amounts of fat, and a high intake of fat, particularly saturated fat, has been associated with unfavorable outcomes and increased mortality (RR = 1.3) among individuals diagnosed with breast cancer [55].

## 6. Conventional Therapy

### 6.1. Adjuvant Therapy

#### 6.1.1. Chemotherapy

**Figure 4:**
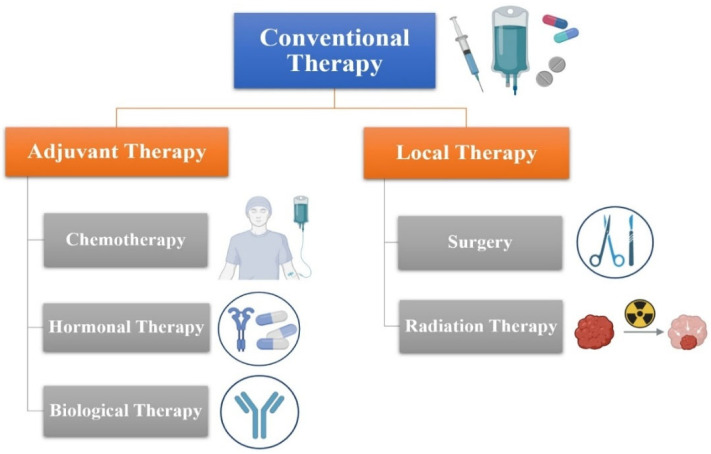
Overview of breast cancer treatment pathways.

For an extended period, breast cancer was perceived as a localized, regional disease that solely impacted the breast and lymphatic system until the 1970s. Adjuvant therapy refers to the administration of systemic treatments when a patient’s previously treated local illness poses a risk of relapse (Figure 4). The benefits of CMF and anthracycline-based treatment were confirmed in a recent meta-analysis that included 100,000 women. Two years later, the percentage of deaths had reduced by 6.2% and 6.5%, respectively [34]. Due to its effectiveness, adjuvant chemotherapy is utilized as neoadjuvant therapy for inoperable patients. Recently, it has become increasingly recognized as a strategy for addressing locally advanced breast cancer. However, there was no difference in disease-free survival or overall survival between neoadjuvant and adjuvant chemotherapy; subset analysis did show that preoperative chemotherapy seemed to benefit younger women more than postmenopausal women [56]. Despite achieving a full radiographic response on MRI, it is essential to completely excise the affected area, as there may still be lingering small islands present. Resistance to a specific chemotherapy regimen often indicates a likelihood of resistance to other treatments, irrespective of the potential advantages of recognizing individuals who exhibit a poor response to a certain therapy.

#### 6.1.2. Hormonal Therapy

Radiation treatment, surgery, or gonadotropin-releasing hormone (GnRH) analogues are the methods that may be used to achieve hormonal ablation. The first step in molecularly targeted cancer treatment is endocrine therapy, which is tailored to breast cancer precisely. Since the ER receptor is present in 70–80% of the population, endocrine treatment is the gold standard for treating ER illness. Nobody has clarified this carry-over impact. Hormonal treatment is beneficial for both node-positive and node-negative breast cancer patients, and it is just as beneficial for younger patients as it is for older ones. According to several studies [57,58,59], aromatase inhibitors (AIs) are recommended for postmenopausal women because they block the synthesis of estrogen in peripheral tissues, where it is produced, and elevated gonadotrophins cannot stimulate a functional ovary.

#### 6.1.3. Endocrine Resistance

An important challenge associated with hormone therapy is endocrine resistance, which occurs when breast cancer cells circumvent estrogen receptor signaling through various mechanisms. The PI3K/Akt/mTOR pathways facilitate certain aspects of these pathways. Cancer cells persist despite hormone blocking because of the hyperactivation of this system. In patients who had progressed on aromatase inhibitors alone, the BOLERO-2 trial demonstrated that the inclusion of everolimus, an mTOR inhibitor, resulted in a significant response rate in the metastatic context [60]. The TAMRAD study indicated that the combination of everolimus and tamoxifen proved advantageous after the failure of aromatase inhibitors [61]. The interaction between the estrogen receptor and HER2 pathways suggests that HER2 overexpression could play a role in developing resistance. Hormonal therapy demonstrates reduced efficacy in individuals with HER2 positivity. The response rate in patients with HER2 positivity has shown improvement when HER2-targeted therapies are used in conjunction with hormone treatments.

#### 6.1.4. Biological and Targeted Therapy

20% of breast cancer cases had overexpressed HER2. Before targeted treatment came along, there was a correlation between HER2 overexpression and a worse chance of survival. Due to its well-established effectiveness in metastatic patients, Trastuzumab, a monoclonal antibody that targets the extracellular region of the HER2 protein, has shown promise when coupled with chemotherapy in many trials. Trastuzumab, when added to chemotherapy for over a year, reduced death risk by one-third and recurrence by half. In clinical practice, a duration of one year remains the standard, despite the lack of clarity regarding the optimal length. Pertuzumab (brand name Perjeta), another monoclonal antibody-based immunotherapeutic drug, inhibits the dimerization of HER2 with itself and other HER family members by binding to a unique site [62]. The internal components of HER1 and HER2 are inhibited by lapatinib, an oral, reversible tyrosine kinase inhibitor. Individuals with advanced disease who had previously had trastuzumab treatment had a longer period before progression [63].

#### 6.1.5. Metastatic Disease

Approximately one-third of individuals receiving treatment for localized breast cancer develop metastatic illness. The average survival time for patients with metastatic disease is under three years, with most succumbing to the illness. Approximately one-third of the locations of initial relapses are attributed to metastases in soft tissue, bone, and visceral organs. However, most patients will exhibit bone involvement at the time of death. Approximately 50% of breast cancer recurrences occur beyond five years after the initial treatment, although they can arise at any time following the primary therapy. The main goals are to uphold the quality of life and to prolong life. Most people in this category receive endocrine treatment and are ER-positive. Surgical removal of localized metastases may be an option for certain individuals [64]. Endocrine treatment is recommended for individuals with ER-positive metastatic illness, but systemic chemotherapy is mostly used for hormone-resistant or ER-negative women.

Metastatic disease symptoms, especially those involving affected bone locations, may be helped by radiation treatment. Although resistance is a factor, the primary and metastatic lesions’ different ER statuses may contribute to the lack of therapeutic response; only 50–70% of tumors identified as ER-positive respond to treatment.

### 6.2. Local Therapy for Nonmetastatic Breast Cancer Surgery

To lessen the functional and cosmetic side effects of localized breast cancer therapy in the long run, surgical care of the disease has come a long way in the last few decades. The traditional approaches, grounded in years of study, involve either excision combined with radiation or a total mastectomy, contingent upon the ability to achieve clear margins (Figure 4). Numerous studies have shown that these two strategies yield similar outcomes regarding overall survival and relapse-free survival rates [65]. If the patient has any of the following: (1) diffuse suspicious microcalcifications seen in breast imaging; (2) positive pathological margins following a lumpectomy; (3) a medical condition that, in nearly all cases, prevents the cosmetic removal of even a small portion of breast tissue; (4) specific collagen-vascular disorders like scleroderma; or (5) a radiation therapy history to the affected breast, then conservative surgery is not an option for you [66]. It is important to treat the breast surgery procedure and the axillary lymph node approach independently. The diagnostic and therapeutic functions of lymph node excision are complementary; the former helps to define the breast cancer’s anatomical extent, while the latter facilitates the removal of cancerous cells. Surgery is decided upon mainly by two criteria: the use of neoadjuvant systemic treatment and the existence of axillary lymph node involvement at the time of diagnosis.

#### Radiation Therapy

Following a mastectomy, radiation treatment for breast cancer may be administered to the chest wall, local lymph nodes, or the whole breast or a part of it following a lumpectomy. One common component of breast-conserving treatment after a lumpectomy is whole-breast radiotherapy [65]. Irrespective of the overall risk associated with breast cancer, the proportional advantage of radiation remained relatively stable, akin to that of adjuvant systemic therapy. As a result, the absolute benefits were larger for those with higher-risk diseases, whereas the mortality benefit confidence interval for those with low-risk node-negative tumors was zero. The effectiveness of a shortened radiation program after a lumpectomy and how to determine which individuals might benefit from greater doses have been the subjects of investigations. Fifty Gy spread out over twenty-five fractions was the conventional wisdom for radiation after lumpectomy. Recent research, however, suggests that a hypofractionated regimen, about 42.5 Gy over 16 fractions, may produce better esthetic results and is just as effective in reducing the likelihood of local recurrence [67,68]. As a result, hypofractionated radiation treatment for the whole breast is recommended by current standards [66].

### 6.3. Limitations

#### 6.3.1. Therapeutic Medicines

Intravenous or oral administration is the usual method of administering treatment medicines for breast cancer. To achieve a concentration within the tumor mass that could lead to lethal toxicity, these agents must navigate through multiple successive barriers. Cellular drug extrusion mechanisms, the reticuloendothelial system, and epithelial and endothelial membranes are all physiological components that pose problems. Tumor vascular architecture, interstitial pressure gradients, extracellular matrix transport, stromal barriers, and density and specificity of tumor-specific surface receptors are all biophysical obstacles. Furthermore, there are physical obstacles to the absorption of some medications, including progestins, luteinizing hormone-releasing hormone agonists, Capecitabine (brand name Xeloda) and Fulvestrant (brand name Faslodex), via the skin and the digestive system [69].

#### 6.3.2. Clearance of Therapeutics from the Circulation

For medications administered intravenously to reach their intended biological target and start working, they must stay in the body for a long enough time. Nonetheless, various defense mechanisms, typically linked to the reticuloendothelial system, are capable of eliminating foreign substances from the bloodstream, encompassing both individual drug molecules and nanoparticles. Individual drug molecules exhibit a circulation half-life of merely a few minutes, whereas particle formulations, which possess half-lives extending to several hours, can effectively deliver equivalent or greater doses of medications throughout the system. This represents a considerable advantage of nanotechnology compared to conventional treatments, potentially reducing or greatly diminishing the necessity for regular chemotherapy injections. It is also possible to build nano vectors with geometric and physicochemical features that allow them to evade or prevent sequestration. Small capillaries are commonly found in the lungs, with an average diameter ranging from 5 to 8 μm [70]. Consequently, vascular embolization would occur due to rigid particles exceeding 5 μm in size, but this phenomenon would be limited to the smallest capillaries, considering the size of the particles [71]. The interendothelial gap junctions, conversely, are likely to permit the extravasation of particles smaller than 20–30 nm from the systemic circulation [72]. The liver, spleen, and lungs are the primary organs involved in the sequestration and trapping of particles. The liver, with a diameter ranging from 10 to 13 μm, exhibits the highest micro-vascularization in both quantity and density. In the endothelial cells of the sinusoid walls, there exist numerous small pores, measuring between 100 and 300 nm, which serve as the adhesion sites for liver Kupffer cells [73].

#### 6.3.3. Tumor Vascular Architecture

Alterations in vascular structure and blood flow dynamics are acknowledged as key characteristics of metastatic disease [74]. The proliferation of cancer cells exerts pressure on the developing wall, leading to irregularities in arterial diameters [75]. Transcapillary “leaking” serves as a defining characteristic of the irregular structure and organization of cancerous blood vessels, leading to an overall reduction in blood flow throughout the tumor vasculature. Bypassing endothelial barriers may be possible with intrathecal pharmaceutical delivery, which has been associated with increased levels and retention of therapeutic medicines close to the tumor mass while reducing systemic side effects [76]. Unfortunately, many malignancies do not fit these criteria, and intratumoral injections, like those used in gene therapy, have only been utilized in clinical settings where the exact location of the tumor is known and easily accessible. Because cancer endothelium expresses different surface receptor proteins than normal endothelium, the tumor vasculature might be a medication delivery target.

#### 6.3.4. Tumor Interstitial Pressure

Enhanced interstitial fluid pressure (IFP) is a hallmark of the majority of solid tumors, as shown by melanoma, colorectal, breast, and head and neck cancers [77]. The duration of drug retention in tumors and the transcapillary transport are both influenced by increased interstitial fluid pressure (IFP). This presents a challenge to treatment as it diminishes the uptake of medications or therapeutic agents into a tumor. The intratumoral fluid pressure diminishes significantly as one moves from the center of the tumor mass towards its periphery while remaining stable within the necrotic core [78]. An elevated intratumoral fluid pressure reduces the time therapeutic agents stay within the tumor by obstructing their infiltration into the tumor tissue and driving them back into the circulatory system. Increased interstitial pressure within tumors is associated with various factors [79]. Notably, patients with invasive ductal carcinomas demonstrate a markedly elevated IFP of 29 ± 3 (SE) mm Hg. The correlation between IFP and tumor growth is direct [80]. It is noteworthy that paclitaxel improves tumor oxygenation by almost 100% and simultaneously reduces the mean interstitial fluid pressure by 36%. Conversely, the administration of doxorubicin did not produce any noticeable impact on oxygenation or interstitial pressure [81]. An approach to remotely controlled thermal ablation using metal-based biocompatible nanoparticles, such as gold and iron oxide nanoparticles, might facilitate the development of new treatments that go beyond traditional chemotherapy. For laser-induced thermal treatment, a novel class of nanoparticles called gold nanoshells with adjustable optical absorption spectra was created. Gold nanoshells administered systemically to mice with xenograft tumors, in combination with near-infrared therapy, significantly reduced tumor development and extended tumor-free life [82].

#### 6.3.5. Endothelial Cell Barrier on the Vessels

Blood arteries are made up of a single layer of endothelial cells, a continuous basement membrane made of different extracellular matrix components, and pericytes. All organs have endothelial cell linings that provide a semi-permeable barrier between the blood and the interstitial spaces. This configuration could serve as an obstacle for small therapeutic agents such as antibodies and injectable nanoparticles. The blood vessels surrounding tumors undergo notable morphological alterations throughout the process of carcinogenesis, with endothelial cells generating various fenestrations that can vary in size from 200 to 300 nm and, in some instances, reach up to 1200 nm [83]. When it comes to cancer therapy, tiny nanoparticles and untargeted therapeutic chemicals may reach the tumor mass via fenestration holes, which are related to passive targeting in first- and second-generation vectors. Even though this morphological change is crucial for most drug-delivery particles, the fenestra’s pores change in size and position over time. Tumor permeability and pore size are influenced by the surrounding microenvironment; orthotopic tumors are more permeable than subcutaneous tumors. Thus, the goal of third-generation vectors is to create a drug delivery system that is immune to vascular leakiness [84].

#### 6.3.6. Cellular Uptake of Therapeutic Agent

Chemotherapeutic and adjuvant treatment drugs and chemicals with biological activity must bind to specific sites on cancer cell surfaces, in the cytoplasm, or in certain parts of the nuclear structure to have any effect. The capacity to penetrate cells is crucial for the therapeutic benefits of the majority of conventional chemo drugs, including doxorubicin, paclitaxel, and etoposide. This differs from compounds that operate via the cell surface or extracellular elements. The cell membrane functions as a regulatory and protective barrier, controlling the movement of chemicals, proteins, and other essential substances. This mechanism not only safeguards the cell from external influences but also facilitates its operation and survival. Membranes may present additional challenges to the delivery of medications. Some cell types exhibit phagocytic or pinocytic capabilities; they include endothelial cells, fibroblasts, osteoclasts, and pericytes [85]. A mechanism closely related to receptor-mediated endocytosis is pinocytosis, which involves the intake of fluids and solutes. Drug delivery systems utilizing nanotechnology will create an optimal foundation for individualized polychemotherapy while simultaneously altering multiple pathways that aid in tumor survival, as the necessity for personalized treatment to address tumor heterogeneity gains wider acknowledgement.

The structural and biological barriers that limit traditional breast cancer therapies are highlighted by the limitations listed above, which include dose-limiting systemic toxicity of chemotherapeutic and endocrine agents, rapid renal and hepatic clearance, heterogeneous tumor vascularization, elevated interstitial fluid pressure, endothelial transport restrictions, and inefficient intracellular drug accumulation. Even physiologically tailored medicines still require sustained therapeutic concentrations, sufficient tissue penetration, and receptor accessibility, all of which are impacted by tumor heterogeneity and changing micro-environment conditions. These difficulties show that, despite their clinical value, traditional approaches are constrained by passive bio-distribution, a lack of spatiotemporal control, and an inadequate capacity to alter the tumor microenvironment. The underlying difficulty of attaining full spatial and molecular precision with conventional methods is highlighted by these constraints. By providing programmable control over bio-distribution, microenvironment response, cellular internalization pathways, and subcellular drug release, nanotechnology fills these deficiencies in addition to improving drug accumulation. Nanosystems change therapy from passive exposure-based treatment to dynamically controlled, multiscale precision intervention by fusing physicochemical engineering and tumor biology. Therefore, the move from traditional modalities to tactics enabled by nanotechnology signifies a change from broad cytotoxicity to mechanism-driven, biologically aware precision oncology.

## 7. Nanotechnology

An early definition of nanotechnology was “the creation of useful materials, devices, and systems utilized to manipulate matter at a small scale, specifically within the range of 1 to 100 nm.” The rapid expansion of nanotechnology’s uses in medicine during the last decade has led to a much broader meaning of the term. Our definition of a nanotechnology tool encompasses four essential components [86]: (1) The device must possess a characteristic size at the nanoscale; (2) it must be artificially created; (3) it must exhibit properties that are distinct due to its nanoscopic dimensions; and (4) its one-of-a-kind conduct needs to be explicable employing suitable mathematical models.

**Figure 5:**
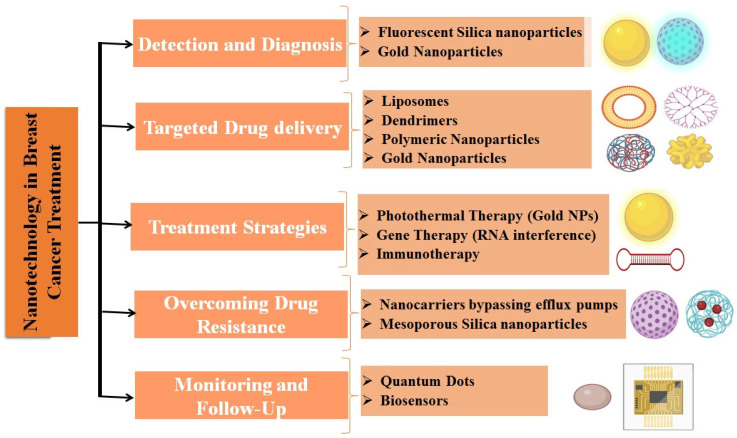
Role of nanotechnology in breast cancer treatment.

Several methods for imaging, monitoring, diagnosing, and delivering chemotherapy medications straight to the location of the cancer have been made possible by nanotechnology and are depicted in Table 1. By overcoming biological barriers and delivering medications with increased efficacy and decreased toxicity, nanoparticles improve anticancer activity. Increased drug penetration and transport are made possible by nanocarriers’ ease of passage through cell membranes and other barriers. Stability, circulation duration, and biocompatibility are among the most crucial aspects to take into account for clinical use, and these can be accomplished by using nanomedicine. To combat a wide variety of illnesses, researchers have created a plethora of nano-delivery devices, each with its own set of material composition and physico-chemical characteristics. Among these, liposomes, polymer-based platforms, dendrimers, gold nanoshells, nanocrystals, carbon-60 fullerenes, silicon- and silica-based nanoparticles, and superparamagnetic nanoparticles are the most extensively studied, as shown in Figure 5 [87,88,89,90,91].

According to recent advancements in NPs, the medicine can be selectively targeted in BC without harming healthy cells or tissues. NPs have unique properties, including high encapsulation efficiency, biocompatibility, ease of manufacturing, and decreased toxicity. One benefit of NPs is that they shield the medication or encapsulated molecules from exposure to the outside world, which shields the medication from harm and keeps it from coming into contact with nearby cells [102]. The multipurpose core–shell nanomedicine can inhibit tumor metastases through photodynamic action, enhancing cytotoxicity. The creation of core–shell nanomedicine used an albumin nanoshell to encase the 20 nm particle diameter of the tyrosine kinase inhibitor Dasatinib. Furthermore, m-tetra (hydroxyphenyl) chlorin, a photosensitizer, is encapsulated inside a PLGA nanocore [100]. To prevent phagocyte breakdown and drug aggregation, polymer micelles were created. When used to treat metastatic breast cancer, the synthetic PTX micelles (Genexol-PM) showed better therapeutic results. For their study, MCF-7 cells and methoxy poly(ethylene glycol)-poly(D,L-lactide) (mPEG-PDLA) copolymer micelles were used to find out what happens when RES and DTX are mixed [103]. The administration of both medications concurrently indicated potential synergistic effects on the breast cancer cells.

In the case of MCF-7 cells, both Ag NPs and Au NPs demonstrated heightened cytotoxic effects [104]. Muthukrishnan et al. developed a leaf extract from Gloriosa superba that contains Ag NP and evaluated its effects on DLA tumor cells [105]. Due to the ability of cancer cells to develop resistance to treatments, a regimen involving multiple medications is mainly formulated for individuals with cancer [106]. One innovative nanocarrier that addresses this limitation found in other delivery systems is mesoporous silica nanoparticles (MSNs). MSNs offer effective intracellular protection because of their restricted range of pore sizes [107]. The surface characteristics of siRNA can be modified to influence its adsorption and release properties. Because of the challenges associated with its incorporation into the core, siRNA is adsorbed onto the surface of nanoparticles and undergoes a cationic transformation. PEI demonstrates greater efficacy compared to organophosphate for modifying the siRNA surface. Nonetheless, these methods are applicable solely for in vitro examinations. Silica nanoparticles are utilized in the fields of tumor imaging and drug delivery. The use of these targeted nanoparticles enables the mapping of sentinel lymph nodes before surgical procedures, facilitating the early identification of breast cancer. The investigation is presently underway in clinical phases I and II [108]. Some of the most significant approaches to cancer therapy have emerged from the rapid development of nanotechnology. When it comes to breast cancer treatment, the most popular nanoparticle kinds are liposomes, micelles, polymeric, solid lipids, and gold nanoparticles.

Small spherical vesicles composed of lipid bilayers and liposomes are utilized in various fields, including drug delivery and cosmetics. Bangham and Horne first created liposomes in 1964. They are round vesicles with an exterior phospholipid membrane that encases water-based molecules. Their sizes vary from around 50 to 200 nm (1). Liposomes allow for the targeted distribution of hydrophilic and hydrophobic medications via their phospholipid layers, which are made of non-immunogenic, biocompatible, and biodegradable materials. Liposomes may concentrate inside tumors because of their small size, which allows them to readily enter vascular holes. It was also shown that using anionic membrane lipids to inhibit P-glycoprotein (P-gp) and using liposomes to load rhodamine 123 as a P-gp substrate increased the retention of rhodamine in MCF-7/P-gp cells. This indicates that P-glycoprotein is active in the transfer of substrates [109].

The structure of the closed, spherical vesicles called liposomes, which were first discovered in 1964 [110], consists of a lipid bilayer encircling an aqueous inner core. Gregoriadis and colleagues first described the use of liposomes in 1971 [111]. Hydrophilic pharmaceuticals, genes, and siRNA (small interfering RNA) may be encapsulated inside the aqueous core of liposomes, which have a diameter ranging from 50 to 100 nm. On the other hand, hydrophobic treatments are integrated into the lipid bilayer. Liposomes offer several advantages, including diminished systemic toxicity, extended circulation time, compatibility with biological systems, and a reduced chance of recognition and clearance by the reticuloendothelial system [112]. The initial nanomedicine to receive FDA approval was Doxil, which consists of DOX encapsulated in PEGylated liposomes. Ovarian cancer, breast cancer that has not responded to other treatments, and Kaposi sarcoma are the main indications for the use of Doxil. In breast cancer patients, the circulatory half-life of Doxil was around 74 h, which is less than free DOX [97]. To specifically target BCSCs and the surface of nucleolin cells, which is overexpressed in TNBC cells, Parvani et al. created F3 peptide-targeted liposomes [97]. A liposomal version of mitoxantrone hydrochloride, an anthraquinone derivative, was clinically assessed in phase II studies for the treatment of recurrent or metastatic breast cancer. To decrease the chances of recurrence and to slow the progression of BC, this liposome was given intravenously to patients.

In cancer therapy, dendrimers stand out as nanocarrier systems, especially when it comes to ligand- or receptor-mediated endocytosis, which allows for targeting and offers several advantages over traditional therapies. Dendrimers are symmetrical nanoparticles with a unique, uniform, monodisperse structure with a diameter between 2 and 10 nm. They are perfect carriers for the targeted delivery of diagnostic and therapeutic substances because of their unique features [113]. A large number of medicines and nucleic acid delivery vehicles were PPI dendrimers produced from branching propylene imine monomers during the BC period [114]. Furthermore, these dendrimers have undergone examination for the treatment of BC following their functionalization with ligands.

Carbon nanomaterials, including graphene, fullerenes, and carbon nanotubes (CNTs), have piqued the curiosity of researchers because of their biocompatibility, chemical functionalization flexibility, and stable physicochemical features. Recent studies indicate that these materials can serve as contrast agents for imaging, diagnosis, and drug localization within tumors, in addition to facilitating controlled delivery of therapeutic agents. Fiorillo et al. indicate that BCSCs exhibit a greater potential for targeting and demonstrate sensitivity to therapy involving graphene oxide-based carbon nanotubes [115]. Graphene oxide nanotubes possess the capability to effectively inhibit the growth of BCSCs across various tumor types, such as glioblastoma in the brain and malignancies affecting the lung, ovary, prostate, breast, and pancreas [116]. To diagnose and treat cancer, the surfaces of fullerenes, carbon nanotubes, and graphenes can be modified with imaging agents and chemotherapeutic drugs [106].

Nanocarriers have the potential to transport various medicinal agents, such as nucleic acids [117]. The method of delivering nucleic acids has demonstrated significant potential in the last two decades, attracting considerable interest as a possible therapeutic approach for various diseases [118]. One of the several isothermal amplification techniques for therapeutic nucleic acids that have been developed to target cancer cells precisely is rolling circle amplification (RCA) [119]. Adding functional groups or successfully integrating with nano-sized entities that have been separated at the atomic level are common ways to chemically modify nucleic acids. The distinct physical and chemical properties of nanomaterials derived from nucleic acids enable advancements in tumor-targeted treatment.

Smaller than 150 nm, gold nanoparticles are non-toxic and have no impact [120]. Recently, gold nanoparticles have gained interest as potential carriers of anticancer medications because of their compatibility with biological systems [1]. It was noted that gold nanoparticles have several benefits, including photothermal treatment and radiosensitization [121]. The study carried out shows that a lactose derivative may keep phthalocyanine-functionalized gold nanoparticles in water solutions stable and distribute them evenly. To create and modify gold nanoparticles, a monolayer made of zinc phthalocyanine and a lactose derivative was used [122]. Functionalizing phthalocyanine-gold nanoparticles with lactose allowed for the development of water-dispersible nanoparticles that produce singlet oxygen, which, when exposed to radiation, kills cells. In vitro experiments have investigated the potential of lactose-functionalized gold nanoparticles to target the galectin-1 receptor found on the surface of MDA-MB-231 and SK-BR-3 breast cancer cells. Lactose shows promise as a targeted treatment against breast cancer cells overexpressing galactose-binding receptors, according to the data. According to several studies, antibodies may bind to gold nanoparticles, allowing them to target antigens that cancer cells overexpress [123]. The manufacture of chloroquine gold nanoparticles and their effects on MCF-7 breast cancer cells were investigated in vitro, and promising anticancer characteristics were found [124]. The development of inulin-coated gold nanoparticles allowed for the targeted delivery of doxorubicin to breast cancer cells [125]. The anticancer activity on MCF-7 breast cancer cells was enhanced by inulin-coated gold nanoparticles, which tended to aggregate more in cancer cells than in normal cells. Novel nanoparticle-based breast cancer treatments have recently emerged. Drug delivery using nanoparticles has a lot of promise, and they are already seeing widespread use in cancer therapy. Strong stability, excellent encapsulation, and the capacity to include hydrophobic and hydrophilic pharmaceuticals are only a few of the benefits shown by nanoparticles. Furthermore, certain cancer cells may become resistant to the medicine while therapy is ongoing, making the drug delivered by the nanoparticle useless [126]. To enhance the delivery of anticancer drugs to target cells, active targeting ligands have been applied to the surfaces of nanoparticles in recent years [127].

### Limitations of Nanotechnology in Breast Cancer Treatment

The practical application of nanotechnology-based treatments for breast cancer is still limited by a number of biological and safety-related issues, despite promising preclinical results. The heterogeneous and frequently unpredictable increased permeability and retention (EPR) response in human malignancies presents one of the biggest obstacles. The main drawback of nanotherapeutic delivery is that, due to the nonspecific uptake of nanoparticles in healthy organs, it is unable to deliver medications at therapeutic amounts to disease sites. Nanoparticles are sequestered soon after injection by the MPS, which is made up of a system of phagocytic cells in the liver, lymph nodes, and spleen, primarily resident macrophages [128]. Opsonization of nanoparticles, which involves the adsorption of plasma proteins such as serum albumin, apolipoproteins, complement components, and immunoglobulins onto the surface of circulating nanoparticles [129], is the first step in the sequestration process. A number of variables, such as nanoparticle size, surface charge, hydrophobicity, and surface chemistry [130], affect how the protein corona forms around them. Following protein adsorption, the nanoparticles connect to particular phagocyte surface receptors before being ingested, moved to phagosomes, and merged with lysosomes [131]. Opsonization not only increases MPS absorption but also frequently works against active-targeted techniques for nanoparticles because the attached biological corona obscures targeting ligands, thereby lowering specificity. This was clearly shown by Dawson and colleagues [132] in a study where silica nanoparticles functionalized with the glycoprotein transferrin failed to connect to soluble transferrin receptors or equivalent receptors on A549 cells when a protein corona formed.

**Figure 6:**
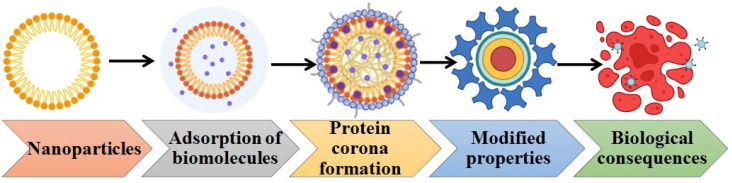
Transformation of nanoparticles in a biological environment.

Since nanoparticles are dynamic entities right after systemic injection, it is necessary to investigate the process underlying the development of protein corona and the potential impacts on the stability, bioavailability, toxicity, and fate of nanoparticles [133,134]. Furthermore, compared to the traditional Cremophor EL formulation of the medication, nanoparticles functionalized with the self-peptide demonstrated increased accumulation in A549 tumors within 10 min of administration. The addition of paclitaxel within the nanobeads resulted in significant tumor shrinking. In an effort to lessen opsonization and subsequent MPS absorption, Tasciotti and colleagues [135] recently created a biomimetic particle covering made of leukocyte cell membranes (Figure 6). Following surface functionalization with leukocytic membranes, protein (IgG and albumin) adsorption to the particle surface decreased by about ten times. Macrophages are appealing targets for therapeutic applications because of their significant role in inflammation and the ensuing disease processes [136]. Wentworth and colleagues [137] used the inflammatory metabolite cholesterol 5,6-secosterol atheronal-B to functionalize the surface of CdSe/ZnS quantum dots. Apolipoprotein B, the protein component of low-density lipoprotein, and other proteins that make up the protein corona underwent structural changes (misfolding/aggregation) as a result of the coating, which improved macrophage uptake of the nanoparticles. By modifying or programming the protein corona, the same technique may be used to improve the uptake of nanoparticles in different cells.

Human breast cancers have varied vascular permeability and dense stromal architecture, which considerably lowers nanoparticle accumulation and therapeutic efficiency, even when nanoformulations show effective passive targeting in animal models. The safety and reproducibility of breast cancer nanotherapy are also impacted by immune system interactions and manufacturing variability, which constitute another significant restriction. Additionally, there have been reports of off-target accumulation in the liver and spleen, cardiotoxicity, and inflammation-related reactions as toxicities linked to nanoparticles (Table 2). Compared to preclinical investigations, clinical trials utilizing liposomal and polymeric nanocarriers have shown variable tumor absorption and limited survival advantages. In order to shorten circulation time and therapeutic exposure, nanoparticles may cause complement activation, hypersensitivity reactions, or quick clearance by the mononuclear phagocyte system.

## 8. Nanorobotics

Cancer cells develop uncontrollably, invading and spreading throughout the body. Since the advent of nanomedicine, which is safer and more effective in overcoming obstacles in tumor therapies, scientists have been working tirelessly to deliver medications to tumor masses.

Chemotherapy often fails due to multidrug resistance (MDR), which is a major obstacle in cancer treatment [146]. The development of resistance in cancer cells to both the original chemotherapeutic agent and subsequent lethal agents with different chemical compositions or action mechanisms after many rounds of therapy is known as MDR [147]. To efficiently destroy malignancies, nanorobots can selectively accumulate in solid tumors when paired with CSC-targeting medications [148].

**Figure 7:**
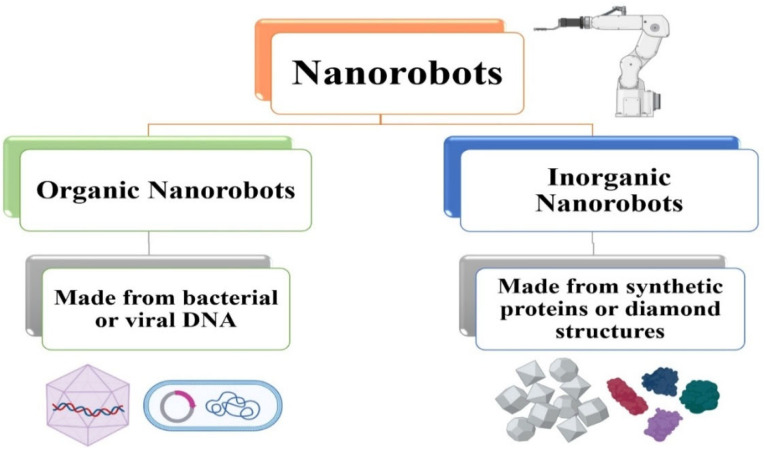
Types of nanorobots (organic and inorganic nanorobots).

Although nanorobots are little machines that may do tasks similar to those of modern machines, the advancement of this new technology has advantages in industry, medicine, and other fields [149]. They can be used to address energy conversion issues with catalytic nanomotors. Current research looks into ways to improve the velocity, force, and lifespan of synthetic nanomotors; some of these nanomotors can move on their own at speeds of up to 100 body lengths per second. Enhancing catalytic nanomotors’ lifetime, velocity, and motion control is crucial for generating electricity in chip microsystems that are driven by autonomous transporters [150]. As depicted in Figure 7, the two categories of nanorobots that are most extensively studied are inorganic and organic. The DNA cells of bacteria and viruses are used to create organic nanorobots, also known as bionanorobots. The organism is less harmed by such a nanorobot. Inorganic nanobots are made of synthetic proteins [151], diamond structures [152], and other materials. These robots are more hazardous; encapsulating the robot can lessen this issue and prevent the body’s defenses from destroying it. This issue has been a significant difficulty for researchers.

**Figure 8:**
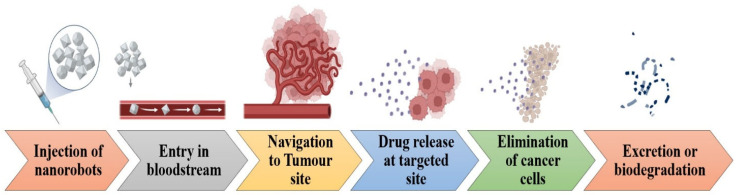
Mechanism of nanorobots for targeted drug delivery. The schematic illustrates the sequential process beginning with the injection of nanorobots and their entry into the bloodstream, followed by active or passive navigation toward the tumor site through biological targeting mechanisms. Upon reaching the targeted tumor microenvironment, nanorobots release therapeutic agents in a controlled manner, leading to selective elimination of cancer cells while minimizing damage to healthy tissues.

In specific studies, proteins are utilized to drive nanomotors capable of transporting large objects. Antibody proteins find applications in DNA hybridization and the development of nanorobots. A nanorobot can be modified with different chemical compounds [153]. A collection of small flat coils was utilized to create a type of inorganic nanorobot featuring a magnetic disk and a lower surface designed for maneuvering into different locations. Magnetic blocks generated by these fields signify possible locations for the nanorobot. DDS, which has a direct impact on specific bodily regions, has been explored within the realm of nanomedicine. Scientists develop devices capable of delivering medications to specific locations while controlling the amount and timing of their release, as shown in Figure 8. This technology offers the advantage of diagnosing and treating illnesses while minimizing harm to healthy cells, thereby decreasing the likelihood of adverse effects [154]. Additionally, it can focus on healing and reconstructive therapies at both the cellular and subcellular levels [155]. Recent developments in drug delivery have led to enhanced quality in targeted drug delivery, utilizing intelligent medications to regulate the release and pinpoint specific cells through self-adhering nanosensors [156].

Some researchers classify nanorobots based on their applications in drug delivery and therapies, as outlined in [156,157]. The individual engaged in the study of chemistry: Medical nanorobots measuring 1–2 μm can contain up to 1 μm^3^ of targeted medication within their tanks. Mechanical systems designed for the sorting of pumps are employed to regulate their operation. The release of weight occurs in either the cytosol, which is the liquid component of a cell’s cytoplasm, or in the extracellular fluid, contingent upon the specific conditions. To make sure they target precisely, they have chemotactic sensors or molecular markers. Glucose and oxygen are drawn from the environment, which includes cytosol, intestinal fluid, and blood, to fuel the onboard system. Centrifuge nanopheresis is capable of removing or recovering the nanorobot once it has completed its designated tasks [152]. The authors discuss microchips that have human molecules layered onto them. Upon detecting an illness, the molecules send an electrical signal from the chip. The advantages include low production costs and ease of manipulation [158].

Due to their capacity to function as bloodborne devices, nanorobots can assist in vital processes in the management of complex diseases, including early diagnosis and targeted drug delivery, as depicted in Table 3 [159]. A nanorobot has the potential to enhance intelligent chemotherapy by facilitating drug delivery and providing a reliable method for early cancer diagnosis. To help with dosing schedules, nanorobots carry drugs into circulation so they may stay there for as long as needed. This capability supports the anticipated pharmacokinetic parameters essential for chemotherapy in cancer treatment [160]. This approach inhibits current extravasations associated with malignancies that are not confined to the reticuloendothelial system and exhibit a significant incidence of degenerative side effects during the chemotherapy process [161]. By integrating chemical nano-biosensors with nanorobot programming, medication delivery and target recognition may be improved. These sensors are capable of detecting different concentrations of beta-catenin and E-cadherin, which serve as medicinal targets during both primary and metastatic stages [162].

Intravenous injection should be used when clinically utilizing nanorobots for diagnosis, treatment, and surgery. As a result, the patient’s bloodstream can receive the nanorobots directly. Chemotherapy pharmacokinetics’ primary cancer treatment cycle consists of absorption, metabolism, and a recovery period before the subsequent chemotherapy treatment. Patients with small tumors often have treatment every two weeks in cycles [170]. With sensors based on proteomics, nanorobots should be able to examine a patient’s bodily fluids and provide a diagnosis in under a week, which is the first time medical diagnostics have been performed. The uptake kinetics of a low molecular weight using a magnetic resonance contrast agent may be used to forecast the delivery of protein medicine to solid tumors [171]. Therefore, a comparable method is helpful to confirm the biosensor activation of in vivo nanorobots through targeted antigen detection [172]. A bacteria-based microrobot, or bacteriobot, is a novel kind of active medication delivery system that was recently disclosed [173]. The work makes use of Salmonella typhimurium, a non-toxic, genetically engineered flagellar bacterium that is drawn to compounds generated by cancer cells. Perrault and Shih of Harvard University’s Wyss Institute for Biologically Inspired Engineering first proposed virus-inspired encapsulated DNA nanostructures as a strategy for medicinal application design [174]. New evidence suggests that nanotechnology and DNA engineering of molecular-scale devices with precise control over shape and site-specific functionalization might pave the way for exciting new developments in nanomedicine. The instability of the biological milieu and the activation of the innate immune system remain, however, obstacles to in vivo deployment. The retention of the medicine in the tumor after targeted delivery by nanorobots across cellular membranes will determine the treatment’s success. To improve the efficacy of chemotherapy for tumors, its composition is affected by drug transfer channels from plasma to tissue [171]. Thus, the primary advantage of using nanorobots to administer cancer medications is the mitigation of chemotherapy-related adverse effects [175]. Hence, anytime the nanorobot detects specified changes in protein gradients, it triggers nanoactuators to regulate medication delivery. The identification of key medicinal targets is intimately tied to changes in chemical and thermal signals.

### Clinical Challenges and Translational Barriers of Nanorobots

By more accurately targeting breast cancer cells, lowering side effects, and enhancing treatment results, nanorobotics have enormous potential to transform the way that breast cancer is treated. Before nanorobots may be widely used for clinical breast cancer treatments, a number of issues need to be resolved [176,177]. A number of technical challenges must be overcome in order to design and operate nanorobots for therapeutic cancer therapies, including creating nanoscale components, managing their motions, and guaranteeing their stability. The accurate control of magnetic nanorobots using externally applied magnetic fields is one of the main problems. It is challenging to attain fine and precise control of nanorobots’ motions due to the intricacy of magnetic fields in small spaces and interference from other electromagnetic waves. This could lead to incorrect tumor site targeting and possible damage to human tissues and organs [178,179]. The biological environment’s interference can significantly impact nanorobots’ accuracy and speed [180]. Interactions between circulating proteins, blood cells, and immune cells and foreign particles can cause nanorobots in blood to move and act more slowly or even be removed [181,182]. There are legitimate worries about the safety and possible negative effects on patients when considering the use of nanorobots in biomedical applications, especially in cancer treatments. In addition to endangering patients, malfunctioning nanorobots may cause unforeseen side effects that make therapy even more difficult [183,184]. A rigorous strategy combining extensive preclinical and clinical testing is necessary to solve these issues and guarantee the safety and effectiveness of nanorobots for cancer treatments. This method will assist in assessing these nanoscale devices’ pharmacokinetics, pharmacodynamics, and biocompatibility in diverse biological settings. The current lack of thorough laws controlling the creation and application of nanorobots may prevent both public and private sector organizations from widely implementing them. The successful integration of nanorobots into clinical cancer treatments depends on the establishment of suitable regulatory frameworks that can effectively address the particular issues presented by nanorobotics while both encouraging innovation and protecting public health [185]. New manufacturing techniques and technologies that can consistently generate complex nanostructures with great precision are needed to mass-produce nanorobots. Larger-scale manufacture of nanorobots may be made possible by developments in fields like self-assembly, 3D printing, and nanolithography [186,187]. Furthermore, production might be further streamlined by incorporating automation and machine learning into manufacturing processes, which would lower human error and boost overall efficiency. These biological constraints directly influence the feasibility of implementing precision nanomedicine in clinical settings. Consequently, even highly engineered precision platforms may fail to activate optimally if adequate intratumoral accumulation is not achieved. Thus, overcoming systemic biological bottlenecks remains a prerequisite for the successful clinical translation of precision-adaptive nanotherapeutics.

## 9. Conclusions

Breast cancer is a major health issue, with special difficulties due to its aggressive nature and limited treatment choices. Although conventional treatments, including radiation, chemotherapy, and surgery, have enhanced patient outcomes, they are often accompanied by restrictions, including systemic toxicity and the development of drug resistance. Employing tailored medication delivery, improved therapeutic efficacy, and minimization of unwanted effects, nanotechnology presents exciting answers to these problems. Overcoming biological constraints, nanoparticle-based platforms have been shown to enable targeted tumor accumulation, extended blood circulation, and regulated medication delivery. Notwithstanding these developments, there are still difficulties, including the requirement of thorough clinical trials to assess the long-term safety and effectiveness of these novel medicines. Future studies should concentrate on optimizing nanoparticle design for personalized medicine, knowledge of the interactions between nanoparticles and biological systems, and investigation of the synergistic possibilities of merging nanotechnology with conventional medical approaches.

## Figures and Tables

**Table 1:** Nanotechnology-based carriers exhibiting promising applications in breast cancer treatment.

Nanomaterials	Components of the Delivery System	Anticancer Drug	Cell Line	Objective	Outcome	Reference
Carbon nanomaterial carrier	Amine-modified PEG-GO nanosheets	Camptothecin analogue	HeLa cells	Enhance the infiltration of fullerenes in breast cancer	Increased surface charge helps penetrate breast cancer cells	[92]
CRISPR NPs	LH NPs	Cas9 protein	MCF-7	For secure methods aimed at the effective and targeted delivery of Cas9 and RNA	In tumors, there is a higher efficacy and inhibition of genes compared to Lipofectamine 2000	[93]
Dendrimers	PAMAM	siMDR1, DOX	MCF-7/ADR	siRNA aimed at the *MDR1* gene to counteract multi-drug resistance (MDR)	Target the *MDR1* gene in MCF-7 cells.	[94]
Inorganic NPs	MSNs	DOX, siRNA	MCF-7/MDR	To overcome breast cancer medication resistance	Effective in vivo tumor growth inhibition and synergy	[95]
Lipid-based drug delivery	SLNs using Compritol 888 ATO	Camptothecin		To enhance encapsulation and cytotoxicity	Improvement in the MCF-7 cell line SLN targeting	[96]
Liposomes	RGD-PEG3400-Mal	F3 peptide, ECO/siβ3		To treat triple-negative breast cancer, give β3 integrin siRNA	Overexpressed nucleolin in triple-negative breast cancer cells should be targeted	[97]
Metallic NPs	PEG tethered AuNPs, PtNPs, PdNPs, AgNPs	DOX, siRNA	MCF-7; MDA-MB-231	BCSC medication delivery, *PAR-1* gene silencing for tumor metastasis	Better DOX delivery to BCSCs, downregulated PAR-1 expression	[98,99]
Polymeric micelles	PLGA, mTHPC	Dasatinib	Metastatic breast cancer cells	Photosensitizer-DAC synergistic cytotoxicity	Src kinase protein disruption disrupts metastatic cancer cell migration	[100]
Polymeric nanoparticles	PEG	DAC	MDA-MB-231	Inhibiting DNA hypermethylation	Overcoming MDA-MB-231 drug resistance	[101]

**Table 2:** Details of clinical trials.

Nano-Formulation	Target	Adverse Effects	Reference
Abraxane (nanoparticle albumin-stabilized paclitaxel)	Assessing the effectiveness of lapatinib plus abraxane as a neoadjuvant treatment for patients with stage I, II, and III breast cancer.	Fatigue, rash, bone pain, anemia, pruritus, diarrhea, neuropathy, fever, mucositis, and vomiting.	[138]
Nab-paclitaxel (nanoparticle albumin-stabilized paclitaxel)	Compare bevacizumab with nab-paclitaxel, doxorubicin, cyclophosphamide, and pegfilgrastim while treating HER2-/NEU-negative breast cancer.	Fatigue, watery eyes, constipation, diarrhea, nausea, anemia, febrile neutropenia, and oral mucositis.	[139]
Nab-paclitaxel (nanoparticle albumin-stabilized paclitaxel)	Assessing the effectiveness of erlotinib plus bevacizumab as a maintenance treatment in women with metastatic breast cancer following nab-paclitaxel and bevacizumab.	Fatigue, neuropathy, infection, and neutropenia.	[139]
Nab-paclitaxel (nanoparticle albumin-stabilized paclitaxel)	Assess nab-paclitaxel + lapatinib’s safety and effectiveness in patients with HER2-positive metastatic breast cancer who have only ever had one chemotherapy regimen.	Acute renal failure, feverish neutropenia, cellulitis, diarrhea, anemia, dehydration, and hypokalemia.	[140]
Nab-paclitaxel (nanoparticle albumin-stabilized paclitaxel)	Evaluating the safety and effectiveness of supplementing women with stage II or III HER2-negative breast cancer with bevacizumab in addition to nab-paclitaxel and carboplatin.	Leukopenia, anemia, thrombocytopenia and neutropenia.	[141]
Nab-paclitaxel (nanoparticle albumin-stabilized paclitaxel)	Investigating the effectiveness of treating metastatic breast cancer with nab-paclitaxel + gemcitabine + bevacizumab.	Anemia, thrombocytopenia, leukopenia, neutropenia, diarrhea, nausea, and nasal hemorrhage.	[142]
Abraxane (nanoparticle albumin-stabilized paclitaxel)	Assess the effectiveness of abraxane, carboplatin, and bevacizumab treatment for TNMBC.	Anemia, thrombocytopenia, neuropathy, exhaustion, constipation, baldness, and anorexia.	[143]
Nanoparticle albumin-stabilized paclitaxel	Examining the effectiveness of doxorubicin hydrochloride + cyclophosphamide + filgrastim in patients with breast cancer who have had surgery in the past, followed by nanoparticle albumin-stabilized paclitaxel with or without trastuzumab.	Fever, dehydration, respiratory issues, gastrointestinal problems, and febrile neutropenia.	[139]
Nab-paclitaxel (nanoparticle albumin-stabilized paclitaxel)	Measuring the effectiveness of carboplatin + nab-paclitaxel + doxorubicin + cyclophosphamide with GM-CSF in patients with 2 cm and/or lymph node-positive breast cancer.	Cardiovascular disease and neutropenic fever	[139]
Abraxane (nanoparticle albumin-stabilized paclitaxel)	Assessing the safety and effectiveness of treating HER2-positive metastatic breast cancer with abraxane + carboplatin + trastuzumab.	Neutropenia, anemia, thrombocytopenia, nausea, constipation, diarrhea, vomiting, exhaustion, neuropathy, and alopecia.	[144]
NK 105 (paclitaxel-incorporating micellar nanoparticle)	Verify the non-inferiority of NK105 to paclitaxel in the treatment of metastatic or recurrent adenocarcinoma of the breast.	Neutropenia, leukopenia, alopecia, neuropathy, rash, nausea, nasopharyngitis, diarrhea, fatigue, stomatitis, nail discoloration, myalgia and dysgeusia.	[145]

**Table 3:** Nanorobot precision surgery applications and investigations.

Nanorobot	Type of Surgery	Objective	Outcomes	References
Catalytic antimicrobial robots (CARs)	Biofilm degradation	This mechanical device swept biofilms over a flat surface, an internal tooth model, and a clogged capillary tube	CARs use iron oxide nanoparticles with dual catalytic-magnetic activity to create bactericidal free radicals, break down the biofilm exopolysaccharide (EPS) matrix	[163]
ExoTIC (Exosome Total Isolation Chip)	Biopsy/sample collection	ExoTIC yields at least 4–1000-fold more EVs than UC and EV-derived protein	Its simplicity, use, and modularity enable high-yield and high-purity EV separation from biofluids	[164]
Magnetic particle drilling	Tissue penetration	Mechanical stimulation of brain tissue by unthreaded micro/nanorobots is shown in this study. After drilling, no particle trace was seen in the brain, confirming past brain slice investigations indicating particles move independently without damaging the brain	Helical dynamic gradients increased particle transport from the nose to the brain compared to linear magnetic gradients alone	[165]
*Magnetospirillum gryphiswaldense*	Biofilm degradation	To mechanically stress E. coli biofilm, a bio-hybrid micro/nanorobot	MSR-1 and drug-loaded mesoporous silica microtubes may create programmable biohybrid micro-swimmers that deliver antibiotics to pathogenic biofilms	[166]
Multifunctional onion-like microtrap vehicle	Biopsy/sample collection	Through regulated chemoattractant and therapeutic drug release, a multifunctional motile microtrap may autonomously attract, trap, and kill microorganisms	Several chemoattractant and therapeutic layers surround a magnesium engine core in the onion-inspired structure. Chemical propulsion depletes the magnesium core, leaving a hollow structure that traps the inner layers	[167]
Needle-type microrobot	Tissue penetration	Paclitaxel (PTX)-loaded MR drug release was characterized to assess targeted drug delivery efficiency. The needle-type MR was shown to target drug administration to MT with varied flow rates in physiological fluidic conditions in vitro	Physical vapor deposition coated Ni/TiO_2_ layers on 3D laser-lithographed MRs. The MR was attached to the target MT using a rotating external magnetic field	[168]
Self-folding thermo-magnetically responsive soft microgrippers	Biopsy/sample collection	Hydrogels like poly (N-isopropyl acrylamide-co-acrylic acid) (pNIPAM-AAc) have low modulus and poor gripping ability for surgical excision	Experiments and modeling were used to develop, construct, and characterize photopatterned, self-folding functional microgrippers	[169]

## Data Availability

No new data were created or analyzed in this study. Data sharing is not applicable to this article.

## Abbreviations

BC—Breast Cancer, TNBC—Triple Negative Breast Cancer, MRP—Multidrug Resistance-associated Protein, EPR—Enhanced Permeation and Retention, CSCs—Cancer Stem Cells, BCSCs—Breast Cancer Stem Cells, GES—Gene Expression Signature, EGFR—Epidermal Growth Factor Receptor, IBC—Inflammatory Breast Cancer, MRI—Magnetic Resonance Imaging, IFP—Interstitial Fluid Pressure, NPs—Nanoparticles, CARs—Catalytic Antimicrobial Robots, ETIC—Exosome Total Isolation Chip, CMOS—Complementary Metal Oxide Semiconductor, HR—Hazard Ratio, HRT—Hormone Replacement Therapy, P-gp—P-glycoprotein, CNTs—Carbon Nanotubes
